# Pre-specified subgroup analyses of a placebo-controlled phase III trial (TEMSO) of oral teriflunomide in relapsing multiple sclerosis

**DOI:** 10.1177/1352458512450354

**Published:** 2012-11

**Authors:** Aaron E Miller, Paul O’Connor, Jerry S Wolinsky, Christian Confavreux, Ludwig Kappos, Tomas P Olsson, Philippe Truffinet, Lin Wang, Laura D’Castro, Giancarlo Comi, Mark S Freedman, for the Teriflunomide Multiple Sclerosis Trial Group

**Affiliations:** 1 Mount Sinai School of Medicine, USA; 2 University of Toronto, Canada; 3 University of Texas Health Science Center, USA; 4 University Claude Bernard Lyon 1, France; 5 University Hospital Basel, Switzerland; 6 Karolinska Institute, Sweden; 7 sanofi-aventis, Chilly-Mazarin, France; 8 sanofi-aventis, New Jersey, USA; 9 Fishawack Communications Ltd, UK; 10 University Vita-Salute San Raffaele, Italy; 11 University of Ottawa, Canada

**Keywords:** Teriflunomide, multiple sclerosis, disease-modifying therapy, subgroup analysis

## Abstract

**Background:**

The Teriflunomide Multiple Sclerosis Oral (TEMSO) trial, a randomized, double-blind, placebo-controlled phase III study, demonstrated that teriflunomide significantly reduced annualized relapse rate (ARR), disease progression and magnetic resonance imaging (MRI) activity, with a favorable safety profile in relapsing multiple sclerosis (RMS) patients.

**Objective:**

The purpose of this study was to report the effects of teriflunomide on ARR and disability progression in pre-specified subgroups.

**Methods:**

RMS patients (*n*=1088) were randomized to placebo or teriflunomide, 7 mg or 14 mg, once daily, for 108 weeks. Subgroup analyses were performed for ARR and disability progression by baseline demographics (gender, race, age), disease characteristics (Expanded Disability Status Scale (EDSS) strata, relapse history, multiple sclerosis (MS) subtype), MRI parameters (gadolinium-enhancing lesions, total lesion volume) and prior use of MS drugs. A generalized estimating equation method and Cox regression model were used to assess consistency of the treatment effect across subgroups, utilizing a treatment-by-subgroup interaction test for each factor separately.

**Results:**

Reductions in ARR and disability progression were consistent across subgroups in favor of teriflunomide, with no treatment-by-subgroup interaction test reaching statistical significance.

**Conclusion:**

The positive effects of teriflunomide were demonstrated consistently across subgroups in TEMSO.

## Introduction

Teriflunomide is a new oral disease-modifying therapy (DMT) currently in phase III development for the treatment of relapsing forms of multiple sclerosis (MS). Teriflunomide selectively and reversibly inhibits the mitochondrial enzyme dihydroorotate dehydrogenase required for de novo pyrimidine synthesis.^1^ As a consequence, teriflunomide blocks the activation and proliferation of stimulated B and T lymphocytes which require de novo synthesis of pyrimidine to expand. Slowly dividing or resting cells, which rely on the salvage pathway for pyrimidine synthesis, are largely unaffected by teriflunomide. The exact mechanism by which teriflunomide exerts its therapeutic effects in MS is not fully understood, but may include a reduced number of activated B and T lymphocytes in the central nervous system (CNS); it is likely that teriflunomide diminishes the number of activated B and T lymphocytes in the periphery that are available to migrate into the CNS.

The Teriflunomide Multiple Sclerosis Oral (TEMSO) trial was the first pivotal phase III study to report from an extensive clinical development program (ClinicalTrials.gov Identifier: NCT00134563).^2^ TEMSO was a randomized, double-blind, placebo-controlled, parallel-group study designed to evaluate the efficacy and safety of teriflunomide in reducing the frequency of relapses and accumulation of physical disability in patients with relapsing multiple sclerosis (RMS) over a two-year treatment period.^2,3^ TEMSO demonstrated that both 7 mg and 14 mg once-daily oral doses of teriflunomide significantly reduced the annualized relapse rate (ARR) (relative risk reductions: 31.2% (*p*=0.0002) and 31.5% (*p*=0.0005)) and 12-week confirmed disability progression (hazard ratio reductions: 23.7% (*p*=0.0835) and 29.8% (*p*=0.0279)) compared with placebo.^2^ Both teriflunomide doses were also superior to placebo on a range of magnetic resonance imaging (MRI) endpoints, including the key MRI endpoint: change from baseline in total lesion volume.^2,4^ TEMSO also demonstrated that teriflunomide was well tolerated with a favorable safety profile, with similar incidences of adverse events, serious adverse events and adverse events leading to discontinuation of study drug across the treatment groups. Among the most common adverse events, with an increased incidence in the teriflunomide groups, were diarrhea, nausea, decreased hair density and elevated alanine aminotransferase (ALT) levels.^2^

The objective of these pre-planned analyses was to determine whether the effects of both doses of teriflunomide on ARR and disability progression were demonstrated consistently in a range of pre-specified patient subgroups from the TEMSO study related to demographic and disease characteristics at baseline.

## Methods

### Patients and procedures

Methodological details of the TEMSO study have been reported in detail elsewhere.^2^ Briefly, eligible patients were 18–55 years of age, met the McDonald criteria for MS diagnosis^5^ and exhibited a relapsing clinical course, with or without progression. Patients were ambulatory (Kurtzke’s Expanded Disability Status Scale (EDSS) score ≤5.5)^6^ with ≥1 relapse in the preceding year (or ≥2 relapses in the previous two years) but with no relapse within 60 days of randomization. Patients were excluded if they had other systemic diseases, were pregnant or planned to conceive during the trial. Patients were stratified by baseline EDSS score (≤3.5 vs >3.5) and were randomized (1:1:1) to three once-daily treatment groups for 108 weeks: placebo; teriflunomide 7 mg; teriflunomide 14 mg. All patients gave written informed consent prior to entering the study.^2^

### Study evaluations

The primary objective of TEMSO was to determine the effect of teriflunomide on ARR, defined as the number of confirmed relapses per patient-year. A relapse was defined as the appearance of a new clinical sign or symptom, or clinical worsening of a previous sign or symptom that had been stable for at least 30 days and persisted for a minimum of 24 hours in the absence of fever. Confirmed relapses required an increase of one point in at least two functional systems, or an increase of two points in at least one functional system (excluding bowel/bladder and cerebral function), or an increase of 0.5 points in EDSS score (1.0 point for EDSS=0) from the previous clinically stable assessment.

The key secondary objective was to determine the effect of teriflunomide on sustained disability progression, as measured by an increase from baseline of at least one point in the EDSS score for at least 12 weeks (or at least 0.5 points for patients with a baseline EDSS score greater than 5.5). The effect of teriflunomide on several MRI parameters,^4^ as well as safety and tolerability, was also assessed.

Pre-specified subgroup analyses were performed for the primary and key secondary clinical endpoints (ARR and 12-week confirmed disability progression) according to baseline demographic features (age, gender, geographical location), clinical disease activity (relapse history, MS subtype), MRI parameters (gadolinium (Gd)-enhancing T1 lesions, total lesion volume) and prior use of other DMTs. The consistency of treatment effect across each subgroup was assessed by a treatment-by-subgroup interaction for each factor separately.

### Subgroup stratifications

ARR and disability progression were analyzed for each treatment group and also by subgroups stratified by various baseline demographic and disease characteristics. Subgroups were selected on the basis of previous studies that have shown them to be potential predictors of clinical outcome in patients with RMS on therapy.^7–9^ Stratifications included baseline demographics (age (<38 years, ≥38 years), gender (male, female) and geographical region (Eastern Europe, Western Europe, Americas)); clinical disease characteristics (EDSS score (≤3.5, >3.5), relapses in the past two years (≤1, 2, 3, ≥4) and MS subtype (secondary progressive/progressive relapsing, relapsing–remitting)); MRI characteristics (number of Gd-enhancing T1 lesions per scan (0, ≥1), total lesion volume (<13 mL, ≥13 mL)); and previous use of disease-modifying MS drugs (yes, no). For these subgroup analyses, the cut-off point for total lesion volume was set to 13 mL and the cut-off point for age was set to 38 years; these thresholds were selected as they lay close to the median total lesion volume and age of all randomized patients at baseline, respectively, at the time of writing the statistical analysis plan.

### Statistical analysis

For each subgroup, ARR was analyzed using a robust Poisson regression model. Response variables were the number of confirmed relapses between randomization and the last dose date; covariates were treatment, region and baseline EDSS strata; and the offset variable was log-transformed standardized exposure duration.

Disability progression was analyzed using a Cox regression model. The dependent variable was time to first disability progression; the test variable was treatment, region and baseline EDSS strata. The Kaplan–Meier method was used to estimate the disability progression rate specific to each arm.

The *p*-value for interaction for ARR was derived using a Poisson model with the number of confirmed relapses between randomization and last dose date as the response variable; treatment, baseline EDSS strata, region, subgroup and treatment-by-subgroup interaction as covariates; and log-transformed standardized study duration as an offset variable. The *p*-value for interaction for 12-week confirmed disability progression was derived from a Cox proportional hazard model with treatment, baseline EDSS strata, region, subgroup and treatment-by-subgroup interaction as covariates.

## Results

### Study disposition

A total of 1088 patients from 127 clinical centers across 21 countries were randomized to receive once-daily doses of placebo, teriflunomide 7 mg or teriflunomide 14 mg for 108 weeks. Of these, 1086 patients were exposed to treatment and formed the modified intention-to-treat population. A total of 869 patients (79.9%) completed the study with similar proportions across the three treatment groups (290 (79.9%), 296 (80.9%) and 283 (78.8%) in the placebo, teriflunomide 7 mg and 14 mg groups, respectively).^2^

### Study population

Baseline patient demographics and disease characteristics of the randomized population separated out according to subgroups under evaluation in this analysis are presented in Table 1. Baseline demographics and disease characteristics were well balanced across the different treatment groups, and reflect a typical population of patients with RMS. Thus, the study population was predominantly female (72.2%) and Caucasian (97.5%) with a mean age of 38 years. The majority of patients had EDSS scores below 3.5, with two or fewer relapses in the two years prior to entry into the study. Most patients had a relapsing–remitting type of disease, but 8.6% of the population had secondary progressive MS with relapses or progressive relapsing MS. In terms of baseline MRI activity, the median total lesion volume was approximately 13 mL, with 36.2% of patients having at least one Gd-enhancing T1 lesion at baseline. Finally, the majority (73.0%) of patients had not received any DMTs within two years prior to randomization; of the 294 patients who had received prior therapy, the distributions of patients within each type of previous treatment was similar across treatment groups.

**Table 1.** Baseline patient demographics according to subgroups under evaluation (randomized population).

	Placebo (*n*=363)	Teriflunomide 7 mg (*n*=366)	Teriflunomide 14 mg (*n*=359)
***Patient demographics***			
**Gender**			
Male	88 (24.2)	111 (30.3)	104 (29.0)
Female	275 (75.8)	255 (69.7)	255 (71.0)
**Age**			
<38 years	156 (43.0)	171 (46.7)	174 (48.5)
≥38 years	207 (57.0)	195 (53.3)	185 (51.5)
**Region**			
Eastern Europe	114 (31.4)	116 (31.7)	108 (30.1)
Western Europe	167 (46.0)	167 (45.6)	170 (47.4)
Americas	82 (22.6)	83 (22.7)	81 (22.6)
***Disease characteristics***			
**Expanded Disability Status Scale score**			
≤3.5	281 (77.4)	281 (76.8)	277 (77.2)
>3.5	82 (22.6)	85 (23.2)	82 (22.8)
**Relapses in past two years**			
≤1	71 (19.6)	74 (20.2)	71 (19.8)
2	186 (51.2)	188 (51.4)	192 (53.5)
3	76 (20.9)	64 (17.5)	70 (19.5)
≥4	30 (8.3)	40 (10.9)	26 (7.2)
**MS subtype**			
Relapsing–remitting	329 (90.6)	333 (91.0)	333 (92.8)
Secondary progressive/progressive relapsing	34 (9.4)	33 (9.0)	26 (7.2)
**Prior use of DMT in last two years**			
Yes	90 (24.8)	102 (27.9)	102 (28.4)
No	273 (75.2)	264 (72.1)	257 (71.6)
***MRI characteristics***			
**Gd-enhancing T1 lesions per scan**^a^	*(n=359)*	*(n=360)*	*(n=355)*
0	222 (61.8)	233 (64.7)	230 (64.8)
≥1	137 (38.2)	127 (35.3)	125 (35.2)
**Total lesion volume**	*(n=358)*	*(n=360)*	*(n=355)*
<13 mL	183 (51.1)	170 (47.2)	183 (51.5)
≥13 mL	175 (48.9)	190 (52.8)	172 (48.5)

DMT: disease-modifying therapy; Gd: gadolinium; MRI: magnetic resonance imaging; MS: multiple sclerosis.

All values are *n* (%) in the individual prospectively defined subgroup.

a Data for Gd-enhancing lesions were missing for four patients in the placebo group, six patients in the 7 mg group and four patients in the 14 mg group.

### Effects of teriflunomide: subgroup analyses

**Figure 1.**
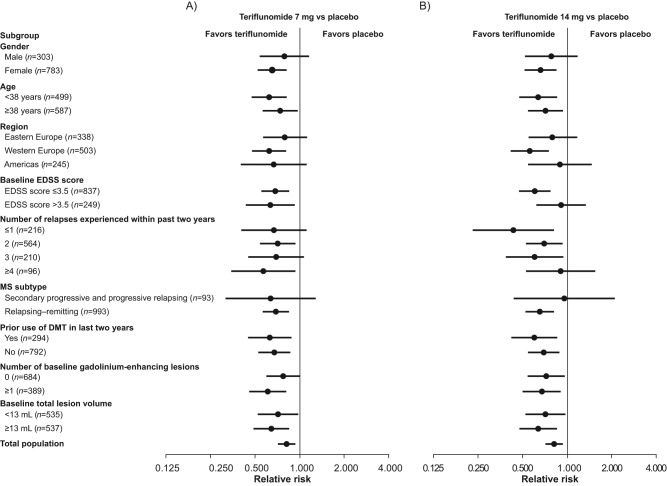
Adjusted annualized relapse rate by patient subgroup. DMT: disease-modifying therapy; EDSS: Expanded Disability Status Scale; MS: multiple sclerosis.

The beneficial effect observed for teriflunomide on ARR was consistent across the different patient subgroups analyzed, whether evaluated according to gender, age, geographic region, baseline EDSS strata, relapse history, MS subtype, MRI parameters or prior use of DMTs. The relative ARR benefit consistently favored both doses of teriflunomide, with no treatment-by-subgroup interaction test reaching statistical significance at the *p*=0.05 level (Table 2). There was, however, a trend towards an interaction for the 14 mg dose and baseline EDSS grouping, with a quantitatively smaller effect on ARR between-group difference in the EDSS score >3.5 stratum compared with the ≤3.5 stratum (*p*=0.07) (Table 2; Figure 1).

**Table 2.** Annualized relapse rate (ARR) by patient subgroup and in total population.

Subgroup	Adjusted ARR^a^ (95% CI)		
	Placebo (*n*=363)	7 mg (*n*=365)	14 mg (*n*=358)
**Gender**			
Male	0.45 (0.33, 0.62)	0.37 (0.28, 0.49)	0.36 (0.26, 0.50)
Female	0.54 (0.46, 0.64)	0.35 (0.29, 0.43)	0.36 (0.29, 0.44)
*p*-value for interaction^b^		0.40	0.62
**Age**			
<38 years	0.73 (0.59, 0.91)	0.45 (0.37, 0.56)	0.47 (0.36, 0.61)
≥38 years	0.43 (0.35, 0.51)	0.31 (0.25, 0.40)	0.31 (0.24, 0.39)
*p*-value for interaction^b^		0.42	0.55
**Region**			
Eastern Europe	0.52 (0.41, 0.67)	0.42 (0.33, 0.53)	0.42 (0.31, 0.55)
Western Europe	0.71 (0.59, 0.86)	0.45 (0.36, 0.55)	0.40 (0.31, 0.51)
Americas	0.31 (0.21, 0.45)	0.21 (0.14, 0.31)	0.27 (0.17, 0.44)
*p*-value for interaction^b^		0.52	0.14
**EDSS score**			
≤3.5	0.50 (0.43, 0.59)	0.35 (0.29, 0.41)	0.30 (0.25, 0.37)
>3.5	0.47 (0.36, 0.63)	0.31 (0.23, 0.42)	0.43 (0.31, 0.60)
*p*-value for interaction^b^		0.81	0.07
**Relapse history**			
≤1	0.38 (0.27, 0.56)	0.26 (0.18, 0.38)	0.17 (0.10, 0.30)
2	0.44 (0.36, 0.55)	0.31 (0.25, 0.39)	0.31 (0.24, 0.40)
3	0.66 (0.51, 0.87)	0.46 (0.32, 0.66)	0.41 (0.28, 0.58)
≥4	1.12 (0.76, 1.63)	0.64 (0.46, 0.88)	1.01 (0.68, 1.52)
*p*-value for interaction^b^		0.88	0.39
**MS subtype**			
SP/PR MS	0.48 (0.21, 1.10)	0.31 (0.13, 0.72)	0.47 (0.21, 1.08)
RRMS	0.54 (0.46, 0.62)	0.37 (0.31, 0.44)	0.36 (0.29, 0.43)
*p*-value for interaction^b^		0.86	0.32
**Previous DMT use**			
Yes	0.78 (0.58, 1.05)	0.50 (0.37, 0.67)	0.47 (0.33, 0.66)
No	0.45 (0.38, 0.54)	0.31 (0.25, 0.38)	0.31 (0.25, 0.40)
*p*-value for interaction^b^		0.85	0.53
**Gd-enhancing lesions**			
0	0.39 (0.32, 0.48)	0.31 (0.25, 0.38)	0.28 (0.22, 0.36)
≥1	0.79 (0.65, 0.96)	0.48 (0.38, 0.60)	0.53 (0.41, 0.69)
*p*-value for interaction^b^		0.17	0.71
**Total lesion volume**			
<13 mL	0.48 (0.38, 0.60)	0.34 (0.27, 0.43)	0.34 (0.26, 0.45)
≥13 mL	0.61 (0.51, 0.74)	0.40 (0.32, 0.49)	0.39 (0.30, 0.50)
*p*-value for interaction^b^		0.63	0.58
**Total population**	**0.54 (0.47, 0.62)**	**0.37 (0.32, 0.43)**	**0.37 (0.31, 0.44)**

ARR: annualized relapse rate; CI: confidence interval; DMT: disease-modifying therapy; EDSS: Expanded Disability Status Scale; Gd: gadolinium; MS: multiple sclerosis; PR: progressive relapsing; RRMS: relapsing–remitting multiple sclerosis; SP: secondary progressive.

a Derived using Poisson model with the total number of confirmed relapses occurring between randomization date and last dose date as the response variable, treatment, EDSS strata at baseline and region as covariates, and log-transformed standardized study duration as an offset variable.

b Derived using Poisson model with the total number of confirmed relapses occurring between randomization date and last dose date as the response variable, treatment, EDSS strata at baseline, region, subgroup and treatment by subgroup interaction as covariates, and log-transformed standardized study duration as an offset variable.

**Figure 2.**
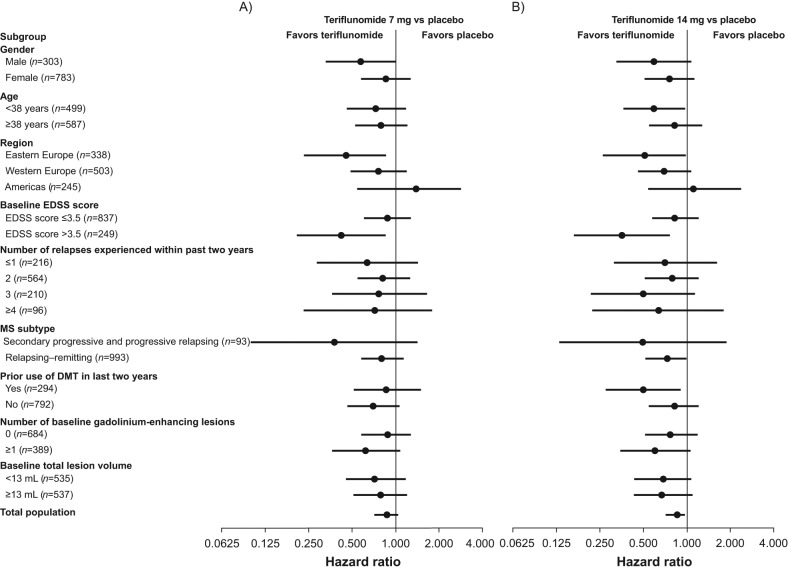
Twelve-week confirmed disability progression by patient subgroup. DMT: disease-modifying therapy; EDSS: Expanded Disability Status Scale; MS: multiple sclerosis.

The effect of teriflunomide on the risk of disability progression was again consistent across each patient subgroup analyzed. Statistical significance was not reached for any treatment-by-subgroup interaction (*p*>0.05). For disability progression, there was a trend towards an interaction for both the 7 mg and 14 mg doses and baseline EDSS grouping, with a quantitatively larger difference in reduction of sustained disease progression for the EDSS score >3.5 stratum as compared with the ≤3.5 stratum (*p*=0.09 and *p*=0.07, respectively) (Table 3; Figure 2).

**Table 3.** Twelve-week confirmed disability progression by patient subgroup and in total population.

Subgroup	Probability of disability progression at week 108^a^ (95% CI)		
	Placebo (*n*=363)	7 mg (*n*=365)	14 mg (*n*=358)
**Gender**			
Male	0.35 (0.24, 0.46)	0.23 (0.14, 0.32)	0.23 (0.14, 0.31)
Female	0.25 (0.20, 0.31)	0.21 (0.16, 0.27)	0.19 (0.14, 0.24)
*p*-value for interaction^b^		0.26	0.51
**Age**			
<38 years	0.28 (0.20, 0.35)	0.22 (0.15, 0.29)	0.18 (0.12, 0.25)
≥38 years	0.27 (0.20, 0.34)	0.21 (0.15, 0.28)	0.22 (0.15, 0.28)
*p*-value for interaction^b^		0.82	0.33
**Region**			
Eastern Europe	0.27 (0.18, 0.35)	0.14 (0.07, 0.21)	0.15 (0.07, 0.22)
Western Europe	0.33 (0.25, 0.41)	0.26 (0.19, 0.33)	0.23 (0.17, 0.30)
Americas	0.17 (0.09, 0.26)	0.24 (0.14, 0.33)	0.21 (0.11, 0.30)
*p*-value for interaction^b^		0.09	0.31
**EDSS score**			
≤3.5	0.26 (0.20, 0.31)	0.23 (0.18, 0.29)	0.22 (0.17, 0.27)
>3.5	0.34 (0.22, 0.46)	0.16 (0.07, 0.25)	0.14 (0.05, 0.22)
*p*-value for interaction^b^		0.09	0.07
**Relapses**			
≤1	0.23 (0.13, 0.34)	0.16 (0.07, 0.25)	0.18 (0.08, 0.28)
2	0.28 (0.21, 0.35)	0.23 (0.17, 0.30)	0.22 (0.16, 0.28)
3	0.26 (0.15, 0.37)	0.21 (0.10, 0.32)	0.15 (0.05, 0.24)
≥4	0.36 (0.16, 0.55)	0.27 (0.12, 0.43)	0.26 (0.08, 0.44)
*p*-value for interaction^b^		0.95	0.80
**MS subtype**			
SP/PR MS	0.32 (0.13, 0.51)	0.13 (0.00, 0.26)	0.19 (0.00, 0.39)
RRMS	0.27 (0.22, 0.32)	0.23 (0.18, 0.27)	0.20 (0.16, 0.25)
*p*-value for interaction^b^		0.29	0.60
**Previous DMT use**			
Yes	0.36 (0.25, 0.47)	0.33 (0.23, 0.43)	0.20 (0.12, 0.29)
No	0.25 (0.19, 0.30)	0.18 (0.13, 0.23)	0.20 (0.15, 0.26)
*p*-value for interaction^b^		0.55	0.15
**Gd-enhancing lesions**			
0	0.26 (0.20, 0.33)	0.23 (0.17, 0.29)	0.20 (0.15, 0.26)
≥1	0.29 (0.20, 0.37)	0.20 (0.12, 0.27)	0.20 (0.12, 0.27)
*p*-value for interaction^b^		0.34	0.50
**Total lesion volume**			
<13 mL	0.26 (0.19, 0.33)	0.19 (0.13, 0.26)	0.19 (0.13, 0.25)
≥13 mL	0.29 (0.21, 0.36)	0.24 (0.17, 0.31)	0.21 (0.14, 0.27)
*p*-value for interaction^b^		0.82	0.94
**Total population**	**0.27 (0.22, 0.32)**	**0.22 (0.17, 0.26)**	**0.20 (0.16, 0.25)**

CI: confidence interval; DMT: disease-modifying therapy; EDSS: Expanded Disability Status Scale; Gd: gadolinium; MS, multiple sclerosis; PR: progressive relapsing; RRMS: relapsing–remitting multiple sclerosis; SP: secondary progressive.

a Derived from Kaplan–Meier estimates.

b Derived from Cox proportional hazard model with treatment, EDSS strata at baseline and region, subgroup and treatment-by-subgroup interaction as covariate.

## Discussion

In the TEMSO study, teriflunomide consistently and significantly reduced the rate of clinical relapse, the risk of disability progression (at the higher dose) and suppressed active inflammatory lesions as visualized on MRI compared with placebo. The benefits of treatment on relapse rate and disability progression were consistent across all pre-defined subgroups whether stratified according to baseline demographic features or disease characteristics.

To contextualize this analysis of the TEMSO data, other subgroup analyses of investigational MS agents were examined. When making comparisons across studies, however, caution should always be exercised owing to differences between study designs and patient populations. A homogeneous treatment effect was seen across all subgroups and baseline demographics analyzed for phase III trials of both oral fingolimod^10^ and cladribine.^11^ A subgroups analysis of a phase II trial with BG-12 also demonstrated homogeneity across all baseline disease characteristics and demographic subgroups investigated.^12^

Teriflunomide is a promising, safe and effective new oral monotherapy for RMS, representing a potential first-line treatment option in this patient population. The beneficial effect of teriflunomide on relapse rate and disease progression was homogeneous across all baseline demographics, clinical and MRI disease characteristics of all the prospectively defined subgroups in the TEMSO study population.
